# Whole-genome sequencing of Chinese centenarians reveals important genetic variants in aging WGS of centenarian for genetic analysis of aging

**DOI:** 10.1186/s40246-020-00271-7

**Published:** 2020-06-10

**Authors:** Shuhua Shen, Chao Li, Luwei Xiao, Xiaoming Wang, Hang Lv, Yuan Shi, Yixue Li, Qi Huang

**Affiliations:** 1 The Center of Health Management and Disease Prevention, The First Affiliated Hospital of Zhejiang Chinese Medical University, Zhejiang, China; 2 Shanghai Institutes for Biological Sciences, Chinese Academy of Sciences, Shanghai, China; 3 Central Laboratory, The First Affiliated Hospital of Zhejiang Chinese Medical University, Zhejiang, China; 4 Shanghai Center for Bioinformation Technology, Shanghai, China

**Keywords:** Longevity, Centenarian, WGS

## Abstract

**Background:**

Genetic research on longevity has provided important insights into the mechanism of aging and aging-related diseases. Pinpointing import genetic variants associated with aging could provide insights for aging research.

**Methods:**

We performed a whole-genome sequencing in 19 centenarians to establish the genetic basis of human longevity.

**Results:**

Using SKAT analysis, we found 41 significantly correlated genes in centenarians as compared to control genomes. Pathway enrichment analysis of these genes showed that immune-related pathways were enriched, suggesting that immune pathways might be critically involved in aging. HLA typing was next performed based on the whole-genome sequencing data obtained. We discovered that several HLA subtypes were significantly overrepresented.

**Conclusions:**

Our study indicated a new mechanism of longevity, suggesting potential genetic variants for further study.

## Introduction

With the development of human genomics research, a large number of studies of the genetics of longevity have been conducted. Scientists from various countries have proposed many different theories concerning the mechanisms of aging from different perspectives, involving oxidative stress, energy metabolism, signal transduction pathways, immune response, etc. [1, 2]. These mechanisms interact with each other and are influenced by heredity to some degree [2, 3]. The identification of longevity-related biological markers is critical to an in-depth understanding of the mechanisms of carrier protection against common disease and/or of the retardation of the process of aging.

Studies revealed from 300 to 750 genes related to longevity that are critically involved in a variety of life activities, such as growth and development, energy metabolism, oxidative stress, genomic stability maintenance, and neurocognition [4]. These candidate genes include mainly APOE, a gene involved in lipoprotein metabolism [5, 6]. Others are those involved in cell cycle regulation, cell growth and signal transduction, the maintenance of genome stability, and the endocrine-related pathway [7–9]. In addition, the candidates for longevity encompass genes related to drug metabolism, the ones involved in protein folding, stabilization, and degradation, as well those related to coagulation and regulation of circulation [10], etc. In most cases, these genes or their polymorphic sites were examined in multiple population replication studies, which discovered certain longevity-associated genes or pathways [4–10].

Besides, longevity is associated with immunity and inflammation [11, 12]. HLA gene, also known as the major histocompatibility complex gene, encodes the major histocompatibility complex (MHC), which is a gene family existing in most vertebrate genomes, closely related to the immune system [13]. Earlier study indicated that HLA may be the genetic basis for the specific response patterns of longevity and longevity immunity [14]. Inflammatory cytokines, such as TNF-α, IL-1β, and IL-6, may be key players [15]. IL-10 limits and terminates the inflammatory response by inhibiting the action of T cells, monocytes, and macrophages, and thus, the genetic variants of this gene may also affect the longevity phenotype [16].

However, at present, most of the investigations on longevity factors of centenarians are performed on a small number of candidate miRNAs, single tissues, or single samples, and only few studies have systematically conducted analyses of multiple tissues and copies at the whole-genome level [17–19].

Based on the results of previous cohort study, in this study, we aimed to use genome-wide sequencing technology to conduct genome-wide association studies and analysis of centenarians. Our findings would facilitate a more accurate focus on the most important genetic basis and molecular mechanisms associated with longevity. The conclusions of this study can serve as the basis for the public efforts towards the extension of the length of life. Moreover, they will provide a scientific reference for further clinical research on disease treatment and overall health care promotion.

## Results

### SKAT analysis revealed significantly correlated genes in aging

The sequencing platform Illumina XTen (Illumina, San Diego, CA, USA) was used for sequencing of the entire genome of 19 centenarians at an average depth of 30×. The sequencing quality metrics are provided in Supplementary Table 1. Baseline information of the centenarians is shown in Table 1. The identified variants were annotated, and non-synonymous variants affecting gene function were selected for association analysis.

**Table 1:** Characteristics of the centenarians

Age at draw	Sex	BMI	Major age-related diseases	Race
104	Male	22.6	None	Eastern Asiatic Mongoloid
103	Male	21.8	None	Eastern Asiatic Mongoloid
102	Female	20.6	None	Eastern Asiatic Mongoloid
105	Female	19.8	None	Eastern Asiatic Mongoloid
116	Female	27.2	None	Eastern Asiatic Mongoloid
101	Female	22.4	None	Eastern Asiatic Mongoloid
102	Female	20.6	None	Eastern Asiatic Mongoloid
104	Male	18.6	None	Eastern Asiatic Mongoloid
103	Male	21.5	None	Eastern Asiatic Mongoloid
103	Female	20.3	None	Eastern Asiatic Mongoloid
101	Male	24.5	None	Eastern Asiatic Mongoloid
105	Female	22.5	None	Eastern Asiatic Mongoloid
103	Male	21.6	None	Eastern Asiatic Mongoloid
104	Female	20.7	None	Eastern Asiatic Mongoloid
102	Female	19.8	None	Eastern Asiatic Mongoloid
100	Female	22.3	None	Eastern Asiatic Mongoloid
103	Male	21.4	None	Eastern Asiatic Mongoloid
114	Female	20.6	None	Eastern Asiatic Mongoloid
100	Male	26.2	None	Eastern Asiatic Mongoloid

**Fig. 1:**
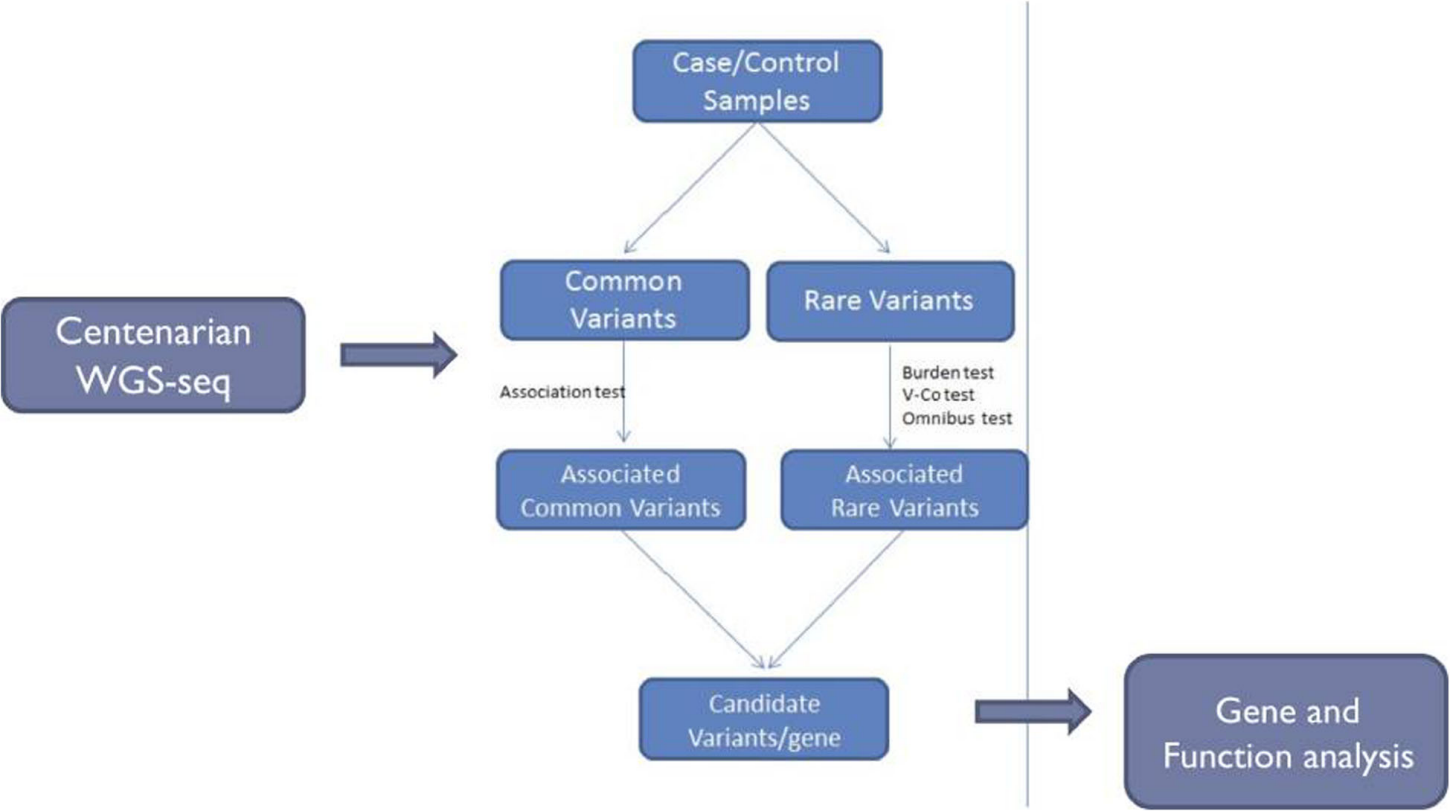
Overall design of the study. WGS were applied to 19 centenarians. Association test was used to select candidate variants/genes. Function analysis was then applied

The experiment design is shown in Fig. 1. Association analysis is applied to WGS data to find important gene and pathways. All centenarian and controls were Eastern Asiatic Mongoloids ascertained to be of Chinese descent (Zhejiang Province, Southeast China). Correlative analysis involved mainly association analysis, SKAT, and Burden tests for rare variants. These methods are commonly employed for GWAS research, especially for case/control samples. They are identified by the difference in frequency of occurrence of variants between case and control samples. Variations associated with the phenotype of the disease, generally directed against common variants, are detected using this method, where the frequency of occurrence and the variation contribute to the phenotype of the disease. Annotations and literature surveys can be used for significantly related variants to further determine the effect of related genes and variants on gene function.

Based on the whole-genome sequencing data, this analysis was performed on the variants detected with MAF > 1%. The control sample consisted of selected 1000G East Asian population data, and the total number of the control samples is 208 [20]. PCA analysis was conducted to evaluate the stratification of the case and control group (supplementary Fig 1). For rare variants with a low frequency of variation, analysis using the method Rare variants case/control association test was performed.

A total number of 41 (Supplementary Table 2) significantly correlated genes were obtained through SKAT analysis. The top 10 genes were as follows: **PABPC3**, **BAGE2**, **HLA-DRB1**, **PDE4DIP**, **PADI4**, **CHI3L2**, **MUC17**, **WARS**, **HLA-DRB5**, and **SIRPB1** (Table 2).

**Table 2:** Significantly correlated genes in SKAT analysis

Gene	SKAT_Common.Only	SKAT_Rare.Only	SKAT_all	SKAT_Rare.pro	SKAT_Common.pro	Burden_all	Burden__Rare.pro	Burden__Common.pro
**PABPC3**	1.97E−27	4.57E−32	4.41E−31	2.72E−48	2.26E−51	2.50E−30	1.46E−50	3.13E−51
**BAGE2**	1.70E−27	6.41E−21	8.76E−25	7.30E−36	1.52E−45	8.66E−26	2.20E−37	5.57E−30
**HLA-DRB1**	0.03254211	1.57E−14	2.66E−13	0.029113904	4.24E−30	1.94E−13	1.33E−21	1.37E−20
**PDE4DIP**	4.19E−07	1.71E−13	2.01E−12	1.13E−09	3.32E−32	5.43E−13	1.40E−16	8.32E−16
**PADI4**	0.351394973	4.20E−10	4.84E−07	0.564180913	3.26E−19	2.18E−08	3.17E−10	9.98E−11
**CHI3L2**	0.182676324	2.30E−09	3.99E−09	9.37E−08	2.84E−24	3.99E−06	9.70E−09	6.54E−09
**MUC17**	0.640011254	2.33E−09	4.68E−08	2.45E−08	3.01E−18	2.60E−06	2.54E−10	3.23E−10
**WARS**	0.248268983	2.07E−08	6.87E−06	6.88E−05	1.99E−15	8.07E−07	1.61E−08	2.34E−08
**HLA-DRB5**	0.021102518	1.05E−06	5.24E−07	0.01776692	3.01E−12	8.72E−08	4.27E−11	5.39E−11
**SIRPB1**	0.479393621	2.95E−06	3.42E−06	5.33E−10	3.26E−10	0.001382303	0.000184681	0.000282752

### Immune system-related pathway was significantly enriched

The significant genes were subjected to differential pathway enrichment analysis. Then, MutsigDB was used to enrich the KEGG and Reactome pathways. As can be seen in Table 3, the associated genes were significantly enriched in the pathways related to immune and inflammatory responses, such as those of interferons, antibodies, and immunity.

**Table 3:** Significantly enriched pathway

Gene set name[#Genes(K)]	Description	#Genes in Overlap(k)	*p* value	FDR *q* value
REACTOME_INTERFERON_GAMMA_SIGNALING[63]	Interferon gamma signaling	5	2.61E−09	2.81E−06
KEGG_ANTIGEN_PROCESSING_AND_PRESENTATION[89]	Antigen processing and presentation	5	1.52E−08	8.16E−06
KEGG_ALLOGRAFT_REJECTION[38]	Allograft rejection	4	3.55E−08	1.28E−05
KEGG_GRAFT_VERSUS_HOST_DISEASE[42]	Graft-versus-host disease	4	5.35E−08	1.40E−05
KEGG_TYPE_I_DIABETES_MELLITUS[44]	Type I diabetes mellitus	4	6.51E−08	1.40E−05
KEGG_CELL_ADHESION_MOLECULES_CAMS[134]	Cell adhesion molecules (CAMs)	5	1.19E−07	2.13E−05
KEGG_AUTOIMMUNE_THYROID_DISEASE[53]	Autoimmune thyroid disease	4	1.40E−07	2.15E−05
REACTOME_TRANSLOCATION_OF_ZAP_70_TO_IMMUNOLOGICAL_SYNAPSE[14]	Translocation of ZAP-70 to immunological synapse	3	2.21E−07	2.97E−05
REACTOME_INTERFERON_SIGNALING[159]	Interferon signaling	5	2.78E−07	3.32E−05
REACTOME_PHOSPHORYLATION_OF_CD3_AND_TCR_ZETA_CHAINS[16]	Phosphorylation of CD3 and TCR zeta chains	3	3.39E−07	3.66E−05

### HLA subtypes are correlated with aging

**Fig. 2:**
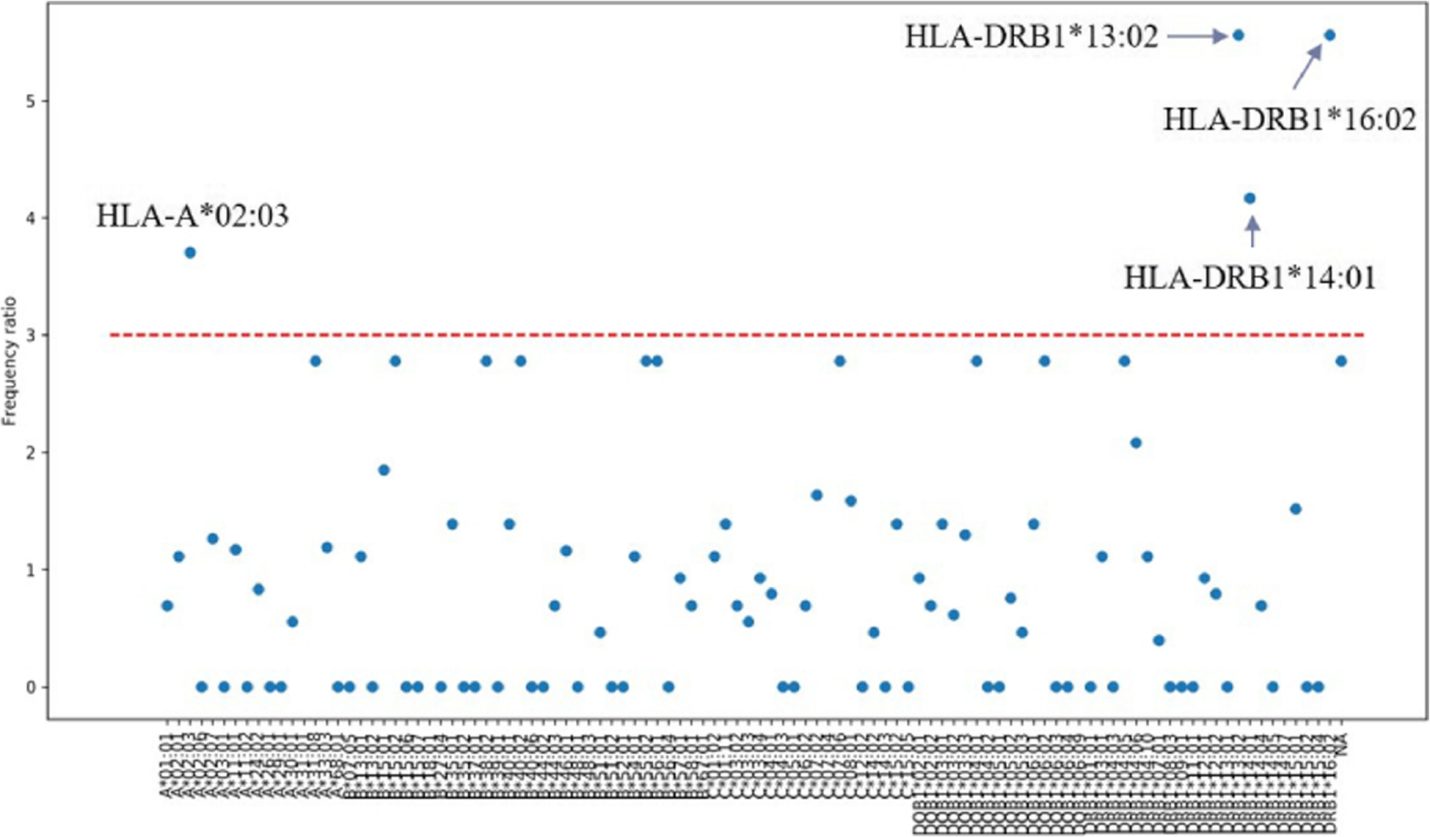
HLA typing’s correlation with the centinarian group. Frequency ratio of every HLA type was plotted. Four HLA types with frequency ratio larger than 3 were marked

Based on the whole-genome sequencing data obtained, HLA-typing was performed. Through the analysis of HLA type distribution, as presented in Tables 3 and 4 and Fig. 2, we found that the type II HLA genes had an important relationship with longevity. Among them, the HLA DRB1 *13:02, HLA DRB1 *14:01, and HLA DRB1 *16:02 were significantly associated with longevity.

**Table 4:** HLA subtype percentage in the case and control groups

HLA type	Ratio	Control_Percentage	Case_Percentage
A*02:03	3.7	0.03	0.11
DRB1*13:02	5.6	0.01	0.05
DRB1*14:01	4.2	0.02	0.08
DRB1*16:02	5.6	0.01	0.05

## Discussion

Researches on the genetic mechanisms of longevity have been conducted from many perspectives, including that on longevity-related genes, variants, and biological pathways [4, 10]. With the advancements in the NGS technology and analysis algorithms, increasingly more longevity-related genetic features could be found and would be useful for the understanding of mechanisms of longevity and related diseases [21, 22].

In our study, we used a small centenarian cohort to establish the association between the genetic variants and longevity. By SKAT analysis, rare variants were found that were related to the longevity phenotype. **HLA-DRB1**, **HLA-DRB5**, and **PDE4 DIP** have been reported to be associated with longevity [23–25]. Importantly, **HLA-DRB1** variants have been specifically reported to have been significantly enriched in a French centenarian study [26]. Further, through the analysis of HLA type distribution, we found several subtypes of **HLA DRB1** which have a closer relationship with longevity. Other significantly related genes that we found in this study, such as **PABPC3**, **BAGE2**, **PADI4**, **CHI3L2**, **MUC17**, **WARS**, and **SIRPB1**, have never been reported before, and their functions deserve further study. Pathway enrichment was performed and showed an important association of the immune-related pathway and the aging process. Previous examinations revealed that immune and inflammatory responses are closely related to ARD (aging-related diseases) [27, 28]. The significant differences in the gene enrichment of the related pathways suggest that a possible longevity mechanism may be associated with protective variants in genes that occur in the related pathways. Here, we have shown that genome-wide data can be further mined as compared with the findings of traditional SNP studies. For example, HLA-type analysis could also be associated with the phenotype, which appears to be a possibility for expanded mining of genome-wide data. Nonetheless, the relatively small sample size limited the power of our findings, and thus further validation by large cohort studies is required.

## Conclusions

In conclusion, the findings of our study provide novel insights into aging mechanisms, suggesting the involvement of several genes, pathways, and specific HLA subtypes that are worth further investigations.

## Materials and methods

### Sample preparation

A random number table was used to randomly collect whole-blood samples from the centenarian cohort in Zhejiang Province, China. Ten percent of centenarians, that is, 19 centenarians, were chosen. All of them were free of major age-related diseases, i.e., cardiovascular or cerebrovascular disease, cancer, dementia, renal or hepatic failure, etc. They were informed about the study and signed a letter of consent, in accordance with the guidelines of the Ethics Committee of the First Affiliated Hospital of Zhejiang Chinese Medical University, Hangzhou, China (2015-KL-008-01). PBMC were isolated to be used for extraction of genomic DNA. Nineteen sex-matched samples with good physical condition were randomly selected from 1000G East Asian population data to serve as a control group [20]. All centenarian and controls were Eastern Asiatic Mongoloids ascertained to be of Chinese descent (Zhejiang Province, Southeast China).

### Library preparation and whole-genome sequencing

Indexed Illumina NGS libraries were prepared from plasma DNA and germline genomic DNA. Next, an NGS library was prepared using the KAPA library preparation kit (Kapa Biosystems). Agencourt AMPure XP beads (Beckman-Coulter) were used to purify the extracted DNA. A 100-fold mole excess of ligation Illumina TruSeq adaptors was used for ligation at 16 °C for 16 h. Size selection of DNA fragments was performed in the 100-μL solution system, and then, the connected fragments were amplified with 500 μm Illumina backbone oligonucleotides for 4–9 rounds of PCR. After that, the DNA fragments were input.

The library concentration was assessed by Qubit and Qpcr. The fragment length was determined using a 2100 Bioanalyzer with a DNA 1000 kit (Agilent). DNA fragments were mixed with HiFi Hot Start Ready Mix (1×), and 2 × 150 bp sequencing of multiple libraries was finally performed with Illumina HiSeq X10.

### Data preprocessing

Paired reads were aligned to the hg19 reference genome using the BWA (V0.7.15-r1140)-mem command [29]. Then, they were sorted and indexed using SAMtools [30]. An in-house Python script was utilized to evaluate the various statistics collected, including mapping statistics, read quality, and panels capture efficiency.

For each sample, the SAMtools pileup function was employed to generate variant candidates among the corresponding sites. We excluded the SNP sites and lower depth sites (≤ eu) among the candidates and removed the reads with low base (< Q30) and mapping qualities (< 40).

### SKAT analysis

The file with the data of the centenarian variants was subjected to SKAT. Functional annotation of the genomic variation of each sample was performed, distinguishing between rare variants and variants affecting the protein function. Next, the influence of the variants in the gene was scored. We used the aforementioned three methods to test candidate genome variants to identify potential rare variants associated with the phenotype. Literature and databases were searched to find how the associated variants affected the biological processes, and speculation on disease mechanisms was carried out.

### Pathway enrichment analysis

The KEGG pathway enrichment was conducted using the DAVID Functional Annotation Bioinformatics Microarray Analysis.

### HLA typing

HLA typing was performed through the HLAscan algorithm [31]. HLAscan started with sequence reads in FASTQ format for mapping to IMGT/HLA data. For targeted sequencing data, sequence reads were used as direct input for HLAscan, whereas for WGS and WES data, we selected reads for HLA genes prior to running the HLAscan. In comparison with the targeted sequencing data, alignment of whole-genome/exome data directly to the IMGT/HLA database may lead to the omission of some HLA reads. Nonetheless, this algorithm was adopted because alignment of HLA reads to the IMGT/HLA database is advantageous in regard to both time and computational processing without loss of predictive accuracy. Initial alignment was performed using BWA-MEM (v0.7.10-r789) with default options. The alignment was the best fit for HLAscan in our investigation, which involved many allele sequences in IMGT/HLA and BWA-MEM. Sequence reads in the BAM file were sorted by reference coordinates using the FixMateInformation function, followed by removal of duplicate reads using MarkDuplicates in the Picard software package (version 1.68) (http://picard.sourceforge.net). Subsequently, identification of indels and re-alignment around these features were performed with the RealignerTargetCreator and IndelRealigner tools, respectively, and base-pair quality scores were recalibrated with BaseRecalibrator and PrintReads using the GATK software (version 3.3.0) [32]. Throughout these processes, sequence reads corresponding to the exonic regions of HLA genes were selected based on an initial alignment generated using GATK with a whole-genome reference (GRCh37.p13). This filtering step does not classify the sequence reads into specific HLA genes.

## Supplementary information

**Additional file 1: Supplementary Figure 1.** PCA plot of the case and control group. PCA plot was made using the SNP genotype of centinarian group and the control group.

**Additional file 2: Supplementary Table 1.** Sequencing quality metrics

**Additional file 3: Supplementary Table 2.** Significantly correlated genes obtained through SKAT analysis.

## Data Availability

Please contact the author for data requests.
