# Supplementary figures and images for: Whole-genome sequencing of Chinese centenarians reveals important genetic variants in aging WGS of centenarian for genetic analysis of aging
Source: Hum Genomics. 2020 Jun 10;14:23. doi: 10.1186/s40246-020-00271-7 (PMC7285530; doi:10.1186/s40246-020-00271-7)

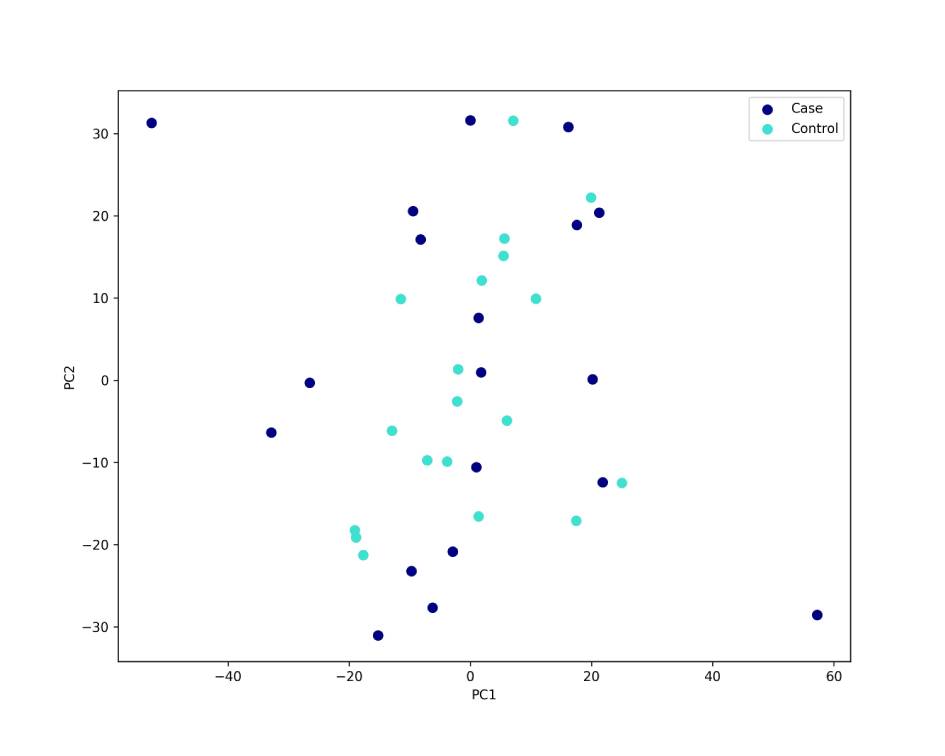

Supplement: Supplementary file 1 — Additional file 1: Supplementary Figure 1. PCA plot of the case and control group. PCA plot was made using the SNP genotype of centinarian group and the control group. [file 40246_2020_271_MOESM1_ESM.jpg]
